# Dentinal Hypersensitivity Treatment Using Diode Laser 980 nm: In Vivo Study

**DOI:** 10.3390/dj7010005

**Published:** 2019-01-09

**Authors:** Marwan El Mobadder, Amaury Namour, Mélanie Namour, Walid Dib, Wassim El Mobadder, Elie Maalouf, Sabine Geerts, Toni Zeinoun, Samir Nammour

**Affiliations:** 1 Department of Dental Science, Faculty of Medicine, University of Liege, 4000 Liege, Belgium; amaurynamour@gmail.com (A.N.); melanienamour@gmail.com (M.N.); walids.dib@gmail.com (W.D.); sabine.geerts@ulg.ac.be (S.G.); S.Namour@ulg.ac.be (S.N.); 2 Department of Endodontics, Faculty of dental medicine, University Saint Joseph, Beirut 1107 2050, Lebanon; wassim.mobader@gmail.com; 3 Department of Periodontology, Faculty of dental medicine, Lebanese University, Beirut 27798, Lebanon; ea.malouf@hotmail.com; 4 Department of Oral Surgery, Faculty of dental medicine, Lebanese University, Beirut 27798, Lebanon; zeinountoni@gmail.com

**Keywords:** dentinal hypersensitivity, diode laser, graphite paste

## Abstract

The discomfort of patients due to dentinal hypersensitivity (DH) is one of the main challenges that dentists face in daily practice. Difficulties in DH treatment gave rise to many protocols which are currently used. The aim of this clinical study is to evaluate the effectiveness of a new protocol on the reduction of dentinal hypersensitivity with diode laser 980 nm and the application of a graphite paste. 184 patients enrolled in the study, the degree of pain was evaluated by visual analog scale (VAS), graphite paste was applied on the exposed dentine before irradiation, the application of diode laser 980 nm with continuous mode, backward motion, tangential incidence of the beam in non-contact mode and a delivery output of 1 W. Fiber’s diameter was 320 μm and total exposure time depended on the time necessary to remove the graphite paste from the teeth. Statistical analyses were performed with Prism 5^®^ software. Pain in post-operative significantly decreased immediately after the treatment. Mean values stayed stable until a 6-month follow-up. The application is considered to be safe with long-term effectiveness.

## 1. Introduction

The discomfort of patients due to dentinal hypersensitivity (DH) is one of the main challenges that dentists face in daily clinical practice [1]. According to Brannstrom’s hydrodynamics theory, when a stimulus is applied to dentine, the fluid inside the tubule will get displaced inwardly and outwardly, causing deformation of the nerve endings at the pulp-dentine interface, and transmitting a painful sensation leading to DH [2]. DH is defined as pain arising from exposed dentin and open tubules, typically in response to thermal, chemical, or mechanical stimuli, and it cannot be explained as arising from any other form of dental defect or pathology [3]. Difficulties in the treatment of the DH gave rise to many protocols and therapeutic procedures which are currently being used for the improvement of DH treatment [4]. Since the mid-1980s, a large number of publications have studied laser technology and its effect on the treatment of DH [5]. The initial results were relatively disappointing; however, advanced technology and the scientific knowledge over the years have improved instrumentation and created new protocols for the treatment [6]. The frequent treatment that has been extensively published is to block the fluid movement in exposed dentinal tubules which will—according to the hydrodynamic theory—reduce the excitability of sensory nerves [7]. A well-known mechanism for treating DH is obliterating or narrowing the dentinal tubules, by inducing hydroxyapatite crystals using laser heat [8]. An adapted exposure to laser energy leads to morphological changes of dentine surface characterized by melted, re-solidified surface and sometimes, in the case of high energy, it leads to the formation of globules and cracks [5].

On the other hand, there have been various reports regarding thermal effects on the dental pulp associated with the use of the dental laser [9,10]. Heat propagation, if not excessive can be tolerated by the dental pulp. However, if the pulp injury is great enough, burn lesions can appear as coagulation necrosis and may provoke intrapulpal abscesses. The literature on laser heating effects on pulpal tissue is somewhat incomplete [11,12]. Some authors have reported various degrees of damage, while others have not investigated the possible secondary effect of laser heat [13]. Nowadays, an increase in pulp temperature of 3 °C is commonly accepted to be the maximum ceiling as not to produce irreversible pulpal damage [14] An ideal treatment for dentin hypersensitivity should not irritate or endanger the pulpal vitality [15,16].

Diode laser 980 nm is in the near infrared position of the electromagnetic spectrum [17] part of the energy is absorbed by the dentinal components provoking melting of the dentin structure [18,19]. These transformations are more intense when higher irradiation parameters are used [14]. Oral tissues contain several chromophores: hemoglobin, melanin, and other pigments [14]. The absorption coefficients of chromophores are variable and dependent on light wavelengths. Diode lasers in the near infrared are more absorbed by melanin and other pigments than by dentin [14].

The absorption coefficients of diode lasers are low in dentin [20]. This low absorption in dentine results in the propagation of the laser beam to the pulpal tissue which will generate heat and may cause undesirable side effects such as hyperemia or irreversible pulpitis [20]. There have been no clinical studies in the literature that uses a pigmented mater on the surface of the dentine in order to prevent the propagation of the beam that may cause pulp injuries. Therefore, in this study, graphite paste (graphite powder mixed to water) was used on the concerned dentinal area before irradiation [14]. The application of graphite paste enhances the absorption of the beam on the dentin surface since—as already mentioned—diode lasers are more absorbed in dark pigments [14]. Consequently, the graphite paste will prevent the propagation of light to the pulp, which will prevent the possible undesirable side effects on the pulpal tissue. The graphite will also provoke an important increase of temperature localized at the dentinal surface to close the dentinal tubules through a melting effect [14]. This sudden and localized rise in temperature will be limited to the dentinal surfaces without important propagation to the pulp [14].

In our study, we based our choice of the irradiation conditions on the results of Umana et al. [14] for the use of safe irradiation conditions for the closure of dentinal tubules. In addition, with the diode laser (980 nm), the same parameters and the application of graphite paste: Temperature increase in pulp due to irradiation was measured in vitro, and a scanning electron microscope (SEM) was performed in order to compare the lased-dentine with non-lased dentine. The aim of this clinical study is to evaluate the clinical effectiveness of diode laser 980 nm with graphite paste applied on the exposed dentinal surface on the reduction of dentinal hypersensitivity. The null hypothesis was that there will be no reduction in post-operative pain, immediately after treatment and for a six-month follow-up for the patients treated with the diode laser (980 nm) and the application of the graphite paste.

## 2. Materials and Methods

The study was conducted in accordance with the Declaration of Helsinki, and the protocol was approved with the identification code of CUMEB/D156/302018 dated on 24 September 2018 by the Ethics Committee of the Lebanese University. All subjects gave their informed consent for inclusion before they participated in the study.

A total number of 184 patients (71% male and 29% female) participated in the study. The inclusion criteria were the following: A minimum of one tooth having DH due to open dentinal tubules with visual analog scale (VAS) > 3.

Exclusion criteria: Patients with teeth showing evidence of irreversible pulpitis or necrosis, carious lesions, defective restorations, facets of attrition, premature contact, cracked enamel, active periodontal disease, use of daily doses of medications, under sedatives, tranquilizers, analgesic, anticonvulsants, and anti-inflammatory medication within 72 h were excluded. All patients who had undergone professional desensitizing therapy during the previous 3 months were also excluded.

For the preoperative pain assessment, a syringe with air/water was placed with a distance of 1 cm perpendicularly to the concerned dentinal tooth and a jet of air/water for only 2 s was applied. Then the patient was invited to measure the pain level using the VAS scale from 0 to 10, where 0 represents “no pain” and 10 represents “greatest pain”.

After pain evaluation, the concerned tooth was ultrasonically cleaned with an ultrasonic scaler to remove any plaque or tartar present. The gingival contour of the concerned tooth was protected by applying a white liquid gingival rubber dam (Discus Dental, LLC Ontario, CA, USA) and then polymerized to avoid any accidental irradiation of the gingiva. The exposed dentine was covered with a graphite powder (ARTGRAF, graphite powder water-soluble Viagro Products) as an enhancer. The graphite was prepared in the same session by mixing distilled water and fine grain (particle size of 5–25 microns). After the protection of the gingiva and the application of the graphite, the exposed dentine was irradiated with diode 980 nm (FONA Laser Sirona Dental Systems GmbH, Bensheim, Germany) as follows: Continuous mode, backward motion, tangential incidence of the beam in non-contact mode (1 mm far from the exposed dentine) and a delivery output of 1 W. The fiber’s diameter was 320 μm and the total exposure time depended on the time necessary to remove the graphite paste from the teeth. The irradiation speed was approximately 1 mm/s (practitioner dependent). All safety measurements for laser irradiation were respected during the study. Practitioners, assistants, and patients wore suitable eyeglasses during the treatment.

The measurements of the pain were performed immediately postoperative, by 3 and 6 months after treatment. 190 patients were enrolled in the study and 184 completed the study. The other 6 patients were unreachable to follow-up.

### 2.1. Statistical Analysis

Statistical analyses were performed with Prism 5^®^ software (GraphPad Software, Inc., San Diego, CA, USA). For the analysis, *P* < 0.05 was considered statistically significant. The confidence level of the study was proposed to be 99% with *P* < 0.001, which is highly significant. Descriptive statistics, including the means and standard deviations, were calculated. One-way Paired ANOVA coupled with a Newman–Keuls Multiple comparison test (PostHoc test) were used.

### 2.2. Pulp Temperature Increase Measurements

Twelve human adult (aged from 18 to 25 years-old) caries-free impacted wisdom teeth were surgically extracted and were kept in balanced salt solution [21] at 4 °C until the experimentation day. Teeth crowns were transversally sectioned at low speed (300 rpm) using a precision sectioning 20 LC diamond blade (Isomet^®^ Low Speed Saw, Buehler^®^ Ltd., Lake Bluff, IL, USA) with the aim to totally expose the dentin. Then they were rinsed with cool water and dried with a five-second air blast [22]. The smear layer was removed by a one-minute application of 18% ethylene diamine tetra-acetic acid (EDTA) (Ultradent Products, Inc., South Jordan, UT, USA). Teeth were rinsed with distilled water.

For each experimentation of pulp temperature rise measurement, the exposed dentinal surfaces were stained with the graphite paste obtained by mixing distilled water and fine grain graphite powder and immediately irradiated by diode laser. At the end of each pulp temperature rise measurement, the exposed dentin was carefully rinsed with distilled water in order to eliminate the residual graphite that could be easily removed. This is due to its particle size being larger than the average diameter of dentinal tubules. Three measurements with graphite paste were performed per tooth at the same irradiation conditions used in vivo for DH treatment. The total time of irradiation for each temperature rise measurement was 10 s.

We followed the protocol used in previous studies for the measurements of pulp temperature increase during laser irradiation [23,24]. The thickness of the dentin between the exposed dentinal surfaces and the pulp roof was 1 mm. The thickness was further confirmed by an X-ray coupled to a millimeter grid. By means of lentulo compactors, the cameral pulps and roots were filled with a thermo-conductor paste (Prosilican thermal compound: warme Leitpaste WPN 10; Austerlitz electronic, Nuremberg 1, Nürnberg, Germany) in order to ensure optimal contact and maximal thermal conduction between the sensor tip of the thermocouple probe and the roof of the cameral pulp. The thermal conductivity of the paste is 0.4 cal·s^−1^·m^−1^·K^−1^. This is comparable to the thermal conductivity of soft tissues (0.2–0.5 cal·s^−1^·m^−1^·K^−1^ depending on hydration) [25].

A type K thermocouple was used (Model TM—946, 4 channels, Lutron, Taiwan), with an accuracy (*precision*) of 0.01 °C. One of the thermocouple probes was placed in close contact with the roof of the cameral pulp [26]. As reference, the second probe was placed at room temperature to compare temperature changes at the roof of the cameral pulp with changes in room temperature.

Once the base pulp temperature became stable after 30 s, we started measuring the temperature variations [26,27]. Pulp temperature was recorded every second for 180 s after the end of the irradiation. Three temperature measurements were recorded for each sample.

The temperature rise (Δ*t*) was calculated as the difference between recorded temperatures at the roof of the cameral pulp (Tcp) before irradiation and the highest registered temperature increase in each experimentation (TRT): Δ*t* = Tcp − TRT [28,29].

The mean of recorded temperatures (Δ*t*) and the standard deviation for each irradiation condition were calculated. Normality tests were carried out using the Kolmogorov and Smirnov test.

### 2.3. Scanning Electron Microscopy (SEM) Analysis

We followed the protocol used in previous studies for the SEM analysis of 6 teeth [8,14]. The irradiated and the unlased dentin (control) treated only with ethylenediaminetetra acetic acid (EDTA) (18%) were dehydrated in blue silicon (with a humidity indicator) at room temperature. At that point, they were attached to aluminum stubs and metalized with a layer of gold (25 nm thick), using vacuum evaporation in a metallizer (model SCD 005, Bautec, Berlin, Germany). The samples were analyzed by SEM (JSM-7610FPlus Schottky Field Emission Scanning Electron Microscope, Japan).

## 3. Results

**Figure 1:**
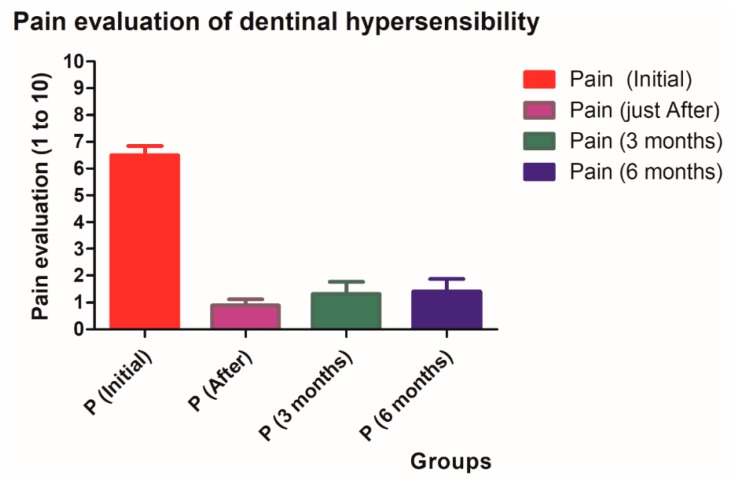
Results of the pain evaluation of dentinal hypersensitivity of different groups with the visual analog scale.

Reduction of dentinal hypersensitivity occurred in all groups in the post-operative period. The pain level averages and standards deviations were: 6.505 ± 1.608 for the initial pain (*P*i), 0.8909 ± 1.045 just after treatment (*P*0), 1.318 ± 2.124 at 3 months after treatment (*P*3) and 1.409 ± 2.153 at 6 months (*P*6) (Table 1, Figure 1).

The pain in the post-operative period significantly decreased immediately after the treatment. Mean values stayed stable until 6 months of follow-up. There was a non-significant increase in pain from *P*3 and *P*6. All groups showed significant differences in pain value average compared to the initial level (Table 2). Out of the 184 patients, no complications or undesired side-effects were reported.

### 3.1. Pulp Temperature Increase Measurements

**Figure 2:**
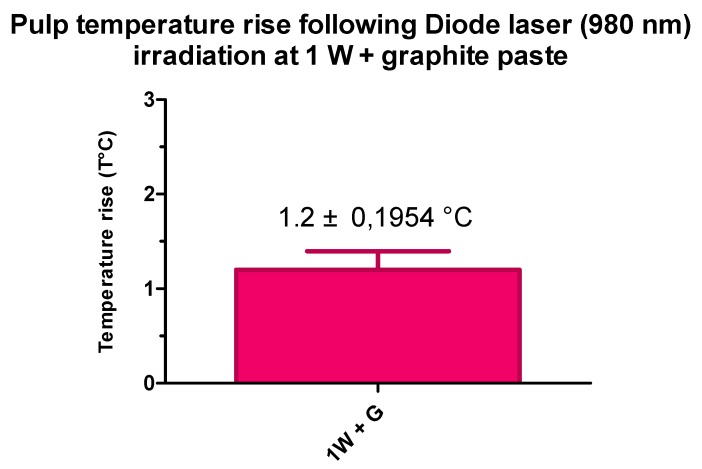
Results of temperature rise following diode laser (980 nm) irradiation at 1 W+ graphite paste. G = Graphite paste.

All samples passed the normality test (Gaussian distribution of values). The mean value of pulp temperature increase was 1.2 °C (±0.1954) with a maximum of 1.500 and a minimum of 0.8000. All values were under the maximum tolerable increase in temperature (Table 3, Figure 2).

### 3.2. Scanning Electron Microscopy (SEM) Analysis

**Figure 3:**
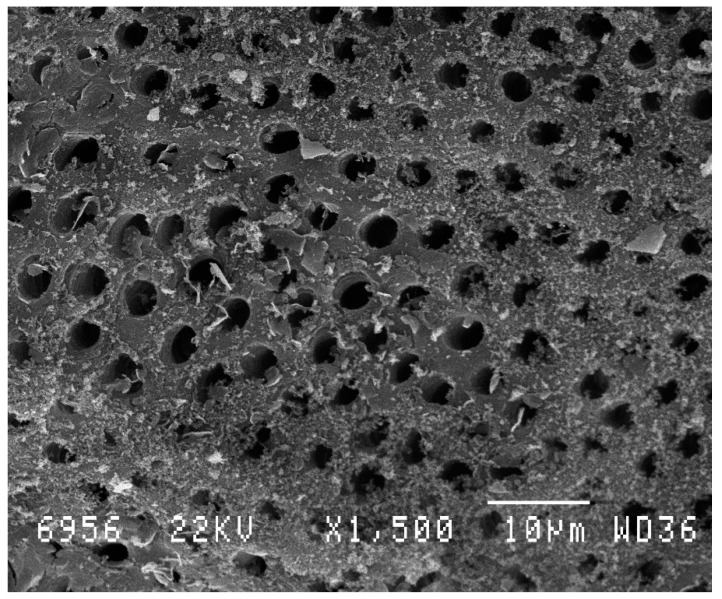
Scanning electron microscopic (SEM) view of unlased dentin (control) treated only with ethylenediaminetetraacetic acid (EDTA) (18%). The dentin is not covered by the smear layer. The tubules are open. Magnification: 1500×.

**Figure 4:**
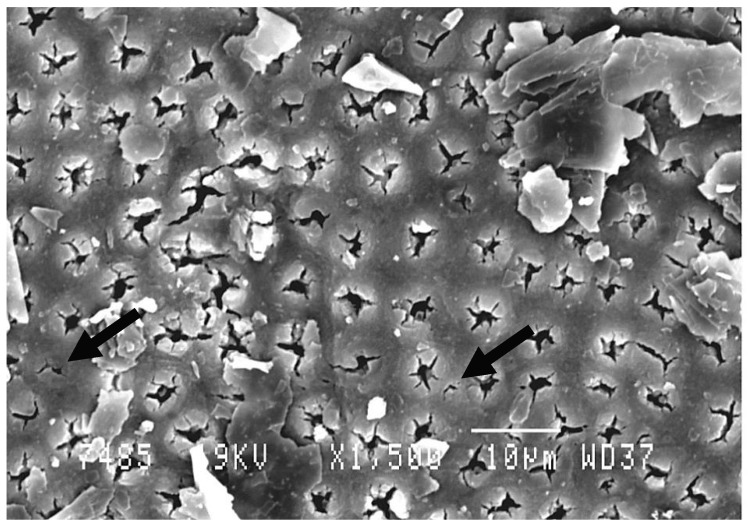
Scanning electron microscopic (SEM) views of treated dentin by diode laser (980 nm) at 1 W. A narrowing of dentinal tubules can be noted. Only a few tubules are completely obliterated. Graphite particles still exist on the dentinal surface (not disintegrated by the laser beam). Magnification: 1500×.

The non-irradiated (control) group presented a normal aspect of open tubules and absence of smear layer (Figure 3). SEM analysis of the irradiated dentin surface showed that a narrowing of the majority of the dentinal tubules was observed. Only a few tubules showed a complete obliteration, and few graphite grains were seen because they were not disintegrated by the laser beam (Figure 4).

## 4. Discussion

According to the results, the null hypothesis was rejected. Dentinal hypersensitivity’s incidence has been increasing due to many factors, and to this day no standard treatment has been available [30,31]. Conventional treatment was based on the use of desensitizing agents fixed on the dentinal surface, but the limitation of this treatment is that these agents do not last; therefore, the effectiveness is just temporary [32]. In this in vivo study, the decrease of DH was statistically significant and with long-term success. The effectiveness of this treatment can be explained by Brannstorm’s hydrodynamic [33] theory. The obliteration of dentinal tubules by laser beam-generated heat inhibits the transmission of the stimulus; therefore, there will be no inward and outward movement of the liquid inside the dentinal tubules, which means there is no pain [33]. In the present in vitro study, it was found that the irradiation with the diode laser 980 nm with the application of the graphite paste do not present any injury to the dental pulp. This is because the increase in temperature with our protocol was less than 3 °C, which is considered as the maximum tolerable increase of temperature [14]. The SEM analysis showed clearly the narrowing of the majority of dentinal tubules after lasing the dentine with the application of the graphite paste. Some graphite pastes were present because the sample was scanned before a proper cleaning of the surface.

Lasers between 800 and 980 nm are poorly absorbed by water and hydroxyapatite [20,34]. This low absorption results in the propagation of the laser beam to the pulpal tissue, which will cause a rise of temperature in the pulp and undesirable side effects such as irreversible pulpitis or hyperemia [35,36]. For this reason, in this in vivo study, graphite paste was used as a pigmented matter on the surface of the dentine before irradiation. The graphite will enhance the superficial absorption of the laser beam which will prevent the propagation of light to the pulp will lead to better dentinal tubules occlusion by increasing the superficial melting effect. The temperature will be sufficient to provoke an immediate superficial dentinal melting, leading to the occlusion or narrowing of dentinal tubules [8]. It is important to note that the graphite paste will only be evaporated and will not be penetrated in the dentinal tubules. In fact, the size of the particles of the graphite paste, 5–25 microns, is greater than the average size of dentinal tubules [8].

Another approach is the tangential angulation of the laser beam that struck the dentinal surface. The aim of this tangential incidence is to avoid a direct pulp exposure by the non-absorbed part of the beam by dentine or by the absorber (graphite paste). The tangential mode was indicated because the reduction of the incident angle towards the refractive angle of the tissue surface increases the potential for true light reflection with an important reduction of pulp absorption of the incident beam. Namour et al. [8] justified these results. They showed that the perpendicular incidence of the laser beams on exposed dentin dramatically increased the pulp temperature even at low output power. In addition, they revealed that with the same power the pulp temperature shows a very low increase in temperature if the irradiation was tangential [8]. Hence, an angulated angle is a safe way to irradiate the hypersensitive dentine because the pulp absorption of the incident beam will be reduced.

In previous studies, authors demonstrated the possibility to occlude dentinal tubules by means of different wavelengths. Ruchi Pandey et al. [37], concluded that low-level laser therapy with diode laser 810 nm at 0.5 W was able to reduce dentinal hypersensitivity [37]. Anely Oliveira Lopes et al., used different protocols combing the laser and desensitizing agents, and revealed that all treatments reduce DH [38]. A meta-analysis concluded that the laser use in reducing DH is efficient except with the Er,Cr:YSSG which did not show any significant difference compared with placebo [38]. Recently, diode laser has been the most used by dentists during day-to-day practice. The literature contains a good amount of studies about this type of laser, particularly its effectiveness against dentinal hypersensitivity [39,40]. The use of the graphite paste and the tangential incidence are new approaches to assure the safety and enhance the positive results of the use of diode laser (980 nm) in dentinal hypersensitivity treatment.

Future studies should be conducted using different laser wavelength with the application of graphite past in order to compare the findings of the diode laser 980 nm used in this study with different wavelengths.

## 5. Conclusions

Within the limitations of this study, diode laser 980 nm coupled to the application of graphite paste, provides a statistically significant decrease in the sensation of pain immediately after one treatment and within a 6-month follow up period. The irradiation condition used in vivo for DH treatment can be considered as safe on pulpal tissue. Therefore, the null hypothesis has been refuted.

## Figures and Tables

**Table 1:** Means and standard deviations for pain values before irradiation (*P* Initial), immediately after irradiation (*P* just after treatment), after 3 months of follow-up (*P* 3 months) and after 6 months of follow-up (*P* 6 months).

	*P* Initial	*P* (Just after Treatment)	*P* 3 Months	*P* 6 Months
Number of values	184	184	184	184
Mean	**6.505**	**0.8909**	**1.318**	**1.409**
Std. Deviation	1.608	1.045	2.124	2.153
Std. Error	0.3429	0.2227	0.4529	0.4590

**Table 2:** Repeated measures ANOVA and Newman–Keuls multiple comparison test between all the groups.

Repeated Measures ANOVA				
*P* value	<0.0001			
*P* value summary	-			
Are means signif. Different? (*P* < 0.05)	Yes			
Number of groups	4			
*F*	139.4			
*R* Squared	0.8691			
Newman–Keuls Multiple Comparison test	Mean Diff.	Q	Significant?	Summary
*P* After vs. *P* Initial	−5.614	24.68	Yes	-
*P* After vs. *P* 3 months	−0.4273	1.878	No	ns
*P* After vs. *P* 6 months	−0.3364	-	No	ns
*P* 6 months vs. *P* Initial	−5.277	23.20	Yes	-
*P* 6 months vs. *P* 3 months	−0.09091	-	No	ns
*P* 3 months vs. *P* Initial	−5.186	22.80	Yes	-

**Table 3:** Pulp temperature increase following diode laser (980 nm) irradiation with graphite paste.

Pulp Temperature at 1 Watt + Graphite	
Number of values	36
Minimum	0.8000 °C
Maximum	1.500 °C
**Mean**	**1.200** °C
Std. Deviation	0.1954
Std. Error	0.05641
Lower 95% CI of mean	1.076
Upper 95% CI of mean	1.324
Kolmogorov Smirnov normality test	
KS distance	0.1667
*P* value	>0.10
Passed normality test (*α* = 0.05)?	Yes
